# Some Reasons Why Physicians Should Attend a Southern Polyclinic

**Published:** 1890-04

**Authors:** 


### SOME REASONS WHY

### Southern Physicians Should Attend a Southern Polyclinic.

It has become qnite a fashion lately to take a post-graduate course; nearly all Texas physicians run off for a session at some Polyclinic; and the fact is very commendable. But we have right here at New Orleans a Polyclinic, which, the JOURNAL learns with much satisfaction, is fully equipped in every department and detail, officered by men of ability and experience, and in every way prepared to bring a practitioner up with the latest accepted teachings in all branches of practice. It is true, the New Orleans polyclinic is fairly patronized by the Texas profession; but not to that extent it should be; for there are many advantages not possessed by other schools, however able and well equipped. This we do not say to the disparagement of the excellent New York Polyclinic, which institution the JOURNAL has often commended, and has helped to build up, nor the excellent Chicago Polyclinic; for, we are on record as having endorsed it, and recommended our readers to go there. But, the amount saved in railroad and hotel expenses alone in going to New Orleans instead of to New York or Philadelphia, will nearly pay for the instruction,—a big item to a poor doctor. The main advantage offered by the New Orleans Polyclinic, however, is in the clinics at the great Charity Hospital. Here, diseases peculiar to the South are mostly seen, and no southern physician need be told that such diseases require quite a different treatment to that adapted to such diseases as are met with in the hospitals of New York or Illinois or Pennsylvania. The New Orleans Polyclinic own their own building, which is within easy access of the great Charity Hospital, where all the clinics are held except the Eye and Ear clinics; these are held only four blocks away at the Eye and Ear Hospital, which, since Prof. DeRoaldes, the Oculist and

Laryngologist, has been secured to the Polyclinic staff, has been thrown open to students. This is an advantage which will be appreciated; for it obviates running from point to point as in New York, for instance, in order to avail of the different clinics. The course of instruction, which begun on 2nd inst., extends over a period of sixty days--two full months, instead of six weeks, as is the case in most Polyclinics.

Now, we are not personally acquainted with a single member of the faculty; we are not carrying their advertisement, and have no interest whatever in the success of the New Orleans Polyclinic; but on principle, we advocate patronizing a Southern school,—all things being equal—in preference to a Northern school; and certainly we do so when there are so many and so manifest advantages. Texas should give it a liberal support.

The following Texas physicians are now in attendance there: Doctors C. A. Danford, Granger; W. W. C. Stirling, Weaver; J. D. Grover, Goldthwaite; W. E. Pennington, Ravenna; G. L. Spurlock, Valley View; J. F. Bertram, Tantis; W. A. Morton, Cottondale; A. M. Denman, Lufton; N. E. Hooper, Mexia; O. H. Coltwell, Dodd City; W. P. Masterson, Corn Hill; C. S. Bobo, Aurona; J. G. Brooking, Georgetown.

